# The endoplasmic reticulum of trypanosomatids: An unrevealed road for chemotherapy

**DOI:** 10.3389/fcimb.2022.1057774

**Published:** 2022-11-10

**Authors:** Jana Messias Sandes, Regina Celia Bressan Queiroz de Figueiredo

**Affiliations:** ^1^ Laboratório de Biologia Celular e Molecular de Patógenos, Departamento de Microbiologia, Instituto Aggeu Magalhães, Recife, Brazil; ^2^ Laboratório de Microscopia Eletrônica, Instituto Keizo Assami, Universidade Federal de Pernambuco, Recife, Brazil

**Keywords:** endoplasmic reticulum, endoplasmic reticulum stress, trypanosomatids, ultrastructure, chemotherapy

## Abstract

The endoplasmic reticulum (ER) of higher eukaryotic cells forms an intricate membranous network that serves as the main processing facility for folding and assembling of secreted and membrane proteins. The ER is a highly dynamic organelle that interacts with other intracellular structures, as well as endosymbiotic pathogenic and non-pathogenic microorganisms. A strict ER quality control (ERQC) must work to ensure that proteins entering the ER are folded and processed correctly. Unfolded or misfolded proteins are usually identified, selected, and addressed to Endoplasmic Reticulum-Associated Degradation (ERAD) complex. Conversely, when there is a large demand for secreted proteins or ER imbalance, the accumulation of unfolded or misfolded proteins activates the Unfold Protein Response (UPR) to restore the ER homeostasis or, in the case of persistent ER stress, induces the cell death. Pathogenic trypanosomatids, such as *Trypanosoma cruzi*, *Trypanosoma brucei* and *Leishmania spp* are the etiological agents of important neglected diseases. These protozoans have a complex life cycle alternating between vertebrate and invertebrate hosts. The ER of trypanosomatids, like those found in higher eukaryotes, is also specialized for secretion, and depends on the ERAD and non-canonical UPR to deal with the ER stress. Here, we reviewed the basic aspects of ER biology, organization, and quality control in trypanosomatids. We also focused on the unusual way by which *T. cruzi*, *T. brucei*, and *Leishmania* spp. respond to ER stress, emphasizing how these parasites’ ER-unrevealed roads might be an attractive target for chemotherapy.

## Introduction

The Trypanosomatidae family comprises a group of parasite protozoans of medical and veterinary interest. These pathogens are found parasitizing vertebrates, insects and even plants (Zuma et al., 2021). Some of them, cause severe diseases in humans, which have in common the fact that they are highly neglected and affecting primarily low-income populations in the development countries, where there is limited investment in prevention, diagnostics, and treatment. Furthermore, these pathologies are highly debilitating, and if they are not treated properly can lead to death. The chemotherapy is still the only alternative to control the diseases caused by these protozoans. However, the currently available drugs used to treat the Chagas disease, sleeping sickness and leishmaniasis have limitations as toxicity, low efficacy, and the resistance of parasites to the treatment is also reported.

**Figure 1:**
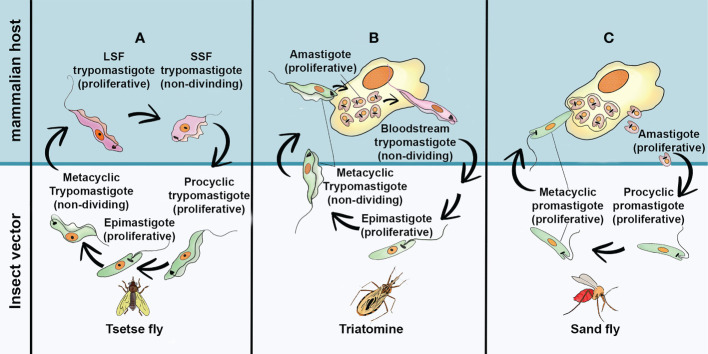
The simplified life cycle of most relevant pathogenic Trypanosomatids: **(A)** *Trypanosoma brucei* **(B)** *Trypanosoma cruzi* and **(C)** *Leishmania* spp. Long Slend Form (SLF); Short Stump Form (SSP). This freehand digital illustration was made using Procreate^®^ and Adobe Photoshop^®^ apps.

*Trypanosoma brucei* is the etiological agent of sleeping sickness or Human African Trypanosomiasis (HAT) and Nagana in cattle. In humans, there are two clinically relevant subspecies of the parasite: *T. b. gambiense* and *T. b. rhodesiensis*. *T. b. gambiense* is present in 24 countries of west and central Africa and is responsible for more than 90% of reported cases of sleeping sickness, whereas *T. b. rhodesiense* is endemic in 13 countries of eastern and southern Africa (Franco et al., 2022). The life cycles of *T. brucei* (**Figure 1A**) may initiate when the infective metacyclic forms, present in the salivary gland of tsetse flies (*Glossina* spp), are transferred to mammalian host during the insect´s blood meal. In the mammalian host this stage differentiates into two main bloodstream forms: the replicative long slender and the short stumpy trypomastigote forms. The first ones can survive and evade the vertebrate host immune response, by expressing antigenically distinct variable surface glycoproteins (VSGs) linked to glycosylphosphatidylinositol anchor (Matthews, 2005; Fall et al., 2022). The short stumpy form is thought to be the only one pre-adapted to survive in the insect vector. During the tsetse bloodmeal, they reach the fly’s midgut, differentiating into the proliferative procyclic form. Then, they cross the peritrophic matrix and migrates to the proventriculus, where they differentiated into epimastigotes. This latter developmental stage can both attach to the salivary gland epithelium as proliferative epimastigotes or differentiate into free swimming cell cycle-arrested metacyclic forms, which is infective to mammalian cells (Schuster et al., 2021). The infection of mammalian host by *T. brucei* is characterized into two clinical stages. In the first one, the parasite multiply in the blood and lymphatic system. The clinical symptoms are usually general in this stage making the diagnosis difficult (Fall et al., 2022). Without prompt treatment the disease can evolve to the second stage, in which the parasites cross the blood–brain barrier causing severe neurological damage and death if untreated (Fall et al., 2022; Kasozi et al, 2022). Currently, there are four approved drugs for treating HAT. Suramin and pentamidine are both used during the first (hemolymphatic) stage, whereas eflornithine and melarsoprol are indicated for the second (meningo-encephalic) stage. The high cost, low oral bioavailability, toxicity, lack of efficacy, and prolonged treatment are the major issues associated with these therapies. The emergence of resistant parasite species for practically all chemically related trypanocidal drugs is also an obstacle to be considered (Kasozi et al., 2022).

*Trypanosoma cruzi* is the causal agent of Chagas Disease (CD), one of the most common and neglected tropical diseases in Latin America, where it is endemic (World Health Organization, 2022a). However, the occurrence of CD in non-endemic regions creates a new epidemiological scenery, which is attributable to population mobility, mainly due to migratory activities, as well as the lack of control on blood banks (Torrecilhas et al, 2020). In Latin America, *T. cruzi* parasites are mainly transmitted by contact with faeces/urine of infected blood-sucking triatomine bugs. However, severe outbreaks due foodborne transmission can sporadically occur (Robertson et al, 2016). According to the World Health Organization (World Health Organization, 2022a), an estimated 6-7 million people are infected with *T. cruzi* worldwide, with about 10,000 deaths every year. The classical life cycle of *T. cruzi* (**Figure 1B**) involves two host and three developmental stages. The infection of a mammalian host begins when the non-dividing metacyclic trypomastigotes, present in the excreta of blood-feeding triatomine vector, penetrate the bite wound and invade a variety of phagocytic and non-phagocytic nucleated cells. During internalization by host cells, the parasite is lodged in a membrane-bound parasitophorous vacuole (PV). Upon entry, the parasites differentiate into small round-shaped amastigote and escape the PV into the cytoplasm. The amastigotes than proliferate by binary fission, until the cells are filled with these replicative forms. At this point, they differentiate into non-replicative trypomastigotes. These forms are extremely motile, and their rapid and intense movement promotes lysis of the host membrane. Once the trypomastigotes are released, they can invade adjacent cells or enter the blood and lymph where they disseminate. Eventually, bloodstream trypomastigote forms, can be taken up by triatomine vector differentiating into proliferative epimastigotes in the vector midgut. The epimastigotes migrate to the vector hindgut and attach to the cuticle to differentiate into infective metacyclic trypomastigote. Although this classical view of *T. cruzi* life cycle is widely accepted, progress has been made regarding the discovery of new morphological and genetically variable evolutive forms of T. *cruzi*, in both insect and mammalian hosts (for a review see, Martín-Escolano et al, 2022). Currently, no safe and efficacious vaccines for this disease are available, and the clinical manage of CD relies on, only two drugs, the nitroheterocyclic compounds benznidazole and nifurtimox. Such treatments are usually indicated for acute cases but their efficacy in adults with chronic CD is lower and variable. Moreover, the high incidence of adverse events with both drugs has hampered their widespread use (Alonso-Veja et al, 2021)

Leishmaniasis is a complex sandfly-borne disease caused by different species of protozoan belonging to the genus *Leishmania.* These illnesses infect 12 million people worldwide and are present in approximately 90 countries, in the tropic and subtropics regions of the Old World (Asia, the Middle East, Africa and southern Europe) and the New World (Mexico, Central America and South America) (Centers for Disease Control and Prevention, 2020; World Health Organization, 2022b). *Leishmania* spp. have two main life cycle morphologies: the intracellular amastigote in the mammalian host and the promastigote in the fly. Following a blood meal, the sandfly ingests macrophages containing *Leishmania* amastigotes. Once inside the sandfly midgut, amastigotes differentiate into procyclic promastigotes. During their migration to the insect salivary gland, procyclic promastigotes undergo a series of morphological differentiation steps into metacyclic promastigotes, which are the mammalian infective forms (**Figure 1C**). The clinical manifestations of leishmaniasis depending on the individual host immune response and the parasite species and are classified in three major forms: cutaneous leishmaniasis (CL), mucocutaneous leishmaniasis (MCL) and visceral leishmaniasis or kala-azar (LV). CL causes skin lesions upon exposure to infection and leaves disfiguring scars and disabilities. This clinical form can progress into its diffuse form where lepromatous lesions are disseminated throughout the skin and are difficult to heal (Menna-Barreto, 2019). In MCL, mucosal membranes are affected, and facial disfiguration are present in almost all cases. In the VL, a strong inflammatory response occurs in the organs, especially the spleen and liver (Menna-Barreto, 2019) resulting in fatal outcome in over 95% of cases, if left untreated. Regardless the clinical form, the pentavalent antimonies are the first choice, but their limited efficacy, severe side effects, inappropriate dosing, long treatment regimens and increased drug resistance constitute the main drawbacks of pentavalent therapy (Menna-Barreto, 2019; Mann et al., 2021). Amphotericin B, in its liposomal form, and Paromomycin have been successfully used as a second choice for parenteral VL treatment, or in the case of CL as topical drug. Pentamidine, used mainly for the treatment of CL, has gained renewed interest as a potential secondary prophylaxis in HIV-coinfected or antimony-resistant VL patients (Dorlo et al, 2012). However, most of these drugs have to be administered parenterally and the cost of treatment is prohibitive for large-scale handling of leishmaniasis in low-income countries. Miltefosine oral administration was firstly introduced in India for the treatment of VL (Dorlo et al, 2012). However, its high cost, teratogenicity and side effects make this compound far from ideal.

During their life cycles, digenetic trypanosomatids are exposed to harsh environmental conditions, undergoing morphological, transcriptomic, proteomic, and metabolic changes with an orchestrated remodelling of organelles. These changes constitute an unpredictably process of adaptation to the microenvironments, in both vertebrate and invertebrate hosts. For example, during the transformation of *T. cruzi* proliferative epimastigotes into infectious metacyclic *T. cruzi* (metacyclogenesis), at the rectal ampulla of the insect vector, these forms are exposure to a poor nutritional environment that increases adenylate cyclase activity and consequently rises intracellular cAMP levels. The increase of cAMP levels stimulates the expression of genes involved in autophagy (Cruz-Saavedra et al, 2020). Like other eukaryotic cells, the ER of trypanosomatids is essential for the adaptation of these protozoans to their lifestyles, sensing and responding to stress insults, controlling the synthesis, processing, and transport of proteins, interacting with other organelles and other microorganisms. Paradoxically the knowledge about the biology of ER in these parasites is still scant. In this review we discussed the morphological and physiological aspects of ER in trypanosomatids such as *T. brucei*, *T. cruzi* and *Leishmania* spp., with the goal of highlighting the relevance of researching the ER for a better understanding of the cell biology of these protozoans. We also discussed how diverse these protozoans have adapted the ER stress response pathways and how these parasites’ ER-unrevealed roads might be an appealing target for chemotherapy.

## Different views of the ER morphology and organization in trypanosomatids

**Figure 2:**
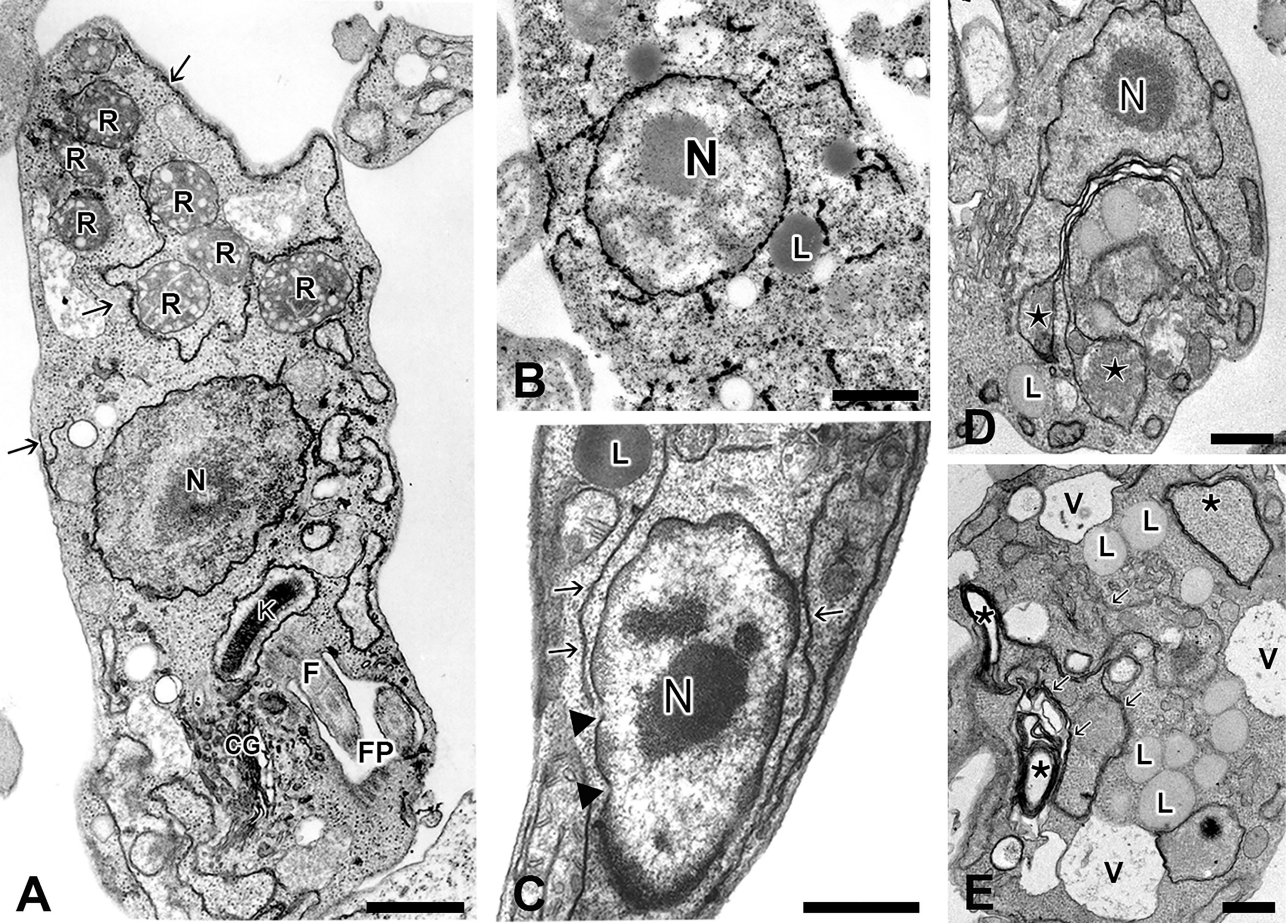
Different morphological views of ER from *Trypanosoma cruzi* and *Leishmania* spp as observed by TEM. **(A)** Epimastigotes of *T. cruzi* processed as routine for TEM, showing scarce profiles of ER (arrows) in close association with plasma membrane and reservosomes (R); **(B)** Detail of an epimastigote submitted to post-fixation with osmium tetroxide/potassium iodide and then processed for TEM, showing electron dense deposits on the nuclear envelope and endoplasmic reticulum (from Figueiredo and Soares, 1995); **(C)** High magnification of a promastigote form of *Leishmania* showing ER profiles (arrows) in the vicinity of nucleus and plasma membrane. Note the presence of well-defined nuclear pores (arrowhead) on the nuclear envelope; **(D–E)** Epimastigotes of *T. cruzi* treated with low **(D)** and high **(E)** concentrations of Tunicamycin (TM), a potent inductor of ER stress, for 72 hours. At low concentration of TM, it is possible to observe an evident increase of ER profiles (from Sandes et al., 2019) compared to control cells. Note a considerable swollen of ER cisternae (stars). **(E)** Detail of an epimastigote treated with high concentration of TM, showing increased number of lipid droplets (L) and vacuoles (V) as well as the appearance of membranous profiles (probably originated from ER) forming myelin-like structures (stars). N, nucleus; GC, Golgi Complex; R, Reservosome; K, kinetoplast; L, lipid inclusions; FP, flagellar pocket, and V, vacuoles. Bars: 0.5 μm.

In higher eukaryotic cells including fungus, animals, and plants, the ER is considered the largest intracellular organelle, consisting of the outer nuclear envelope, and an intricate network of tubules and cisternae, that extend throughout the cytoplasm (Almeida, 2021). Morphologically, the ER of trypanosomatids is a highly dynamic organelle, a mandatory requirement to perform a plethora of essential functions for parasite survival. This organelle undergoes constant structural rearrangements in response to internal and external stimuli. In these parasites the ER is usually continuous with the nuclear envelope (NE) and comprising a connected system of cisternal or tubular membranes distributed throughout the cell and often closely associated with the plasma membrane. The NE has multiple pores that span the inner and outer membranes and are morphologically identical to those found in other eukaryotic cells ( **Figure 2C**). (McConville et al., 2002; Faria, 2021; Zuma et al., 2021). The outer membrane is decorated with ribosomes suggesting that it is a part of o rough ER. However, the borders between rough and smooth ER reticulum, in these protozoans are not well defined and markers of both subdomains are present in the isolated fractions of NE (McConville et al, 2002).

The distribution and abundance of ER in these protozoans can vary enormously depending on the developmental stage, environmental conditions, and the specie of parasites ( **Figure 2**). In epimastigotes of *T. cruzi*, for example, scarce endoplasmic reticulum profiles are found scattered along the cytoplasm (**Figure 2A**), making the ER difficult to be visualized in samples prepared as the routine for transmission electron microscopy. The introduction of osmium tetroxide-potassium iodide methodology during the post-fixation was applied to better visualization of the NE, the ER and the Golgi complex (GC) in epimastigotes forms under transmission electron microscopy (TEM) ( **Figure 2B**). The electron dense staining observed by this methodology is attributed to the reducing environment of these compartments that is able to reduce osmium in the presence of potassium iodide (Figueiredo and Soares, 1995). In *Leishmania* spp. promastigotes this organelle is usually observed closely to the flagellar pocket and GC, and from this region, the ER radiates and occupies the intracellular space (Pimenta and De Souza, 1985; Zuma et al, 2021). However, ER profiles can be also observed in the vicinity of the nucleus in promastigote forms (**Figure 2C**). Incubation of briefly glutaraldehyde-fixed *L. amazonensis* with a cytochemical medium containing glucose 6-phosphate as substrate and lead as capture reagent led to theappearance of dense reaction product, indicative of glucose-6-phosphatase activity in the cisternae of the ER, particularly concentrated in the region near to the parasite´s flagellar pocket (Pimenta and De Souza, 1985). The ER assumes a higher level of organization, not observed in other trypanosomatids, in epimastigotes forms of the insect parasite *Strigomonas culicis* (formerly classified as *Blastocrithidia culicis*), (Soares and de Souza, 1987). The ultra-thin sections observed by TEM showed a smooth reticulum consisting of long cisterna with an electrodense content, which run longitudinally throughout the whole body of the parasite, being mainly concentrated at the cell periphery, immediately below the layer of subpellicular microtubules. On the other hand, rough endoplasmic reticulum was usually observed as stacked cisternae tightly associated to lipid inclusions (Soares and de Souza, 1987). In *T. brucei* ER-localized invariant glycoproteins IGP34, IGP40 and IGP48 are significantly up regulated in the short stumpy bloodstream stage and at least IGP48 are required to maintain the normal architecture of ER (Allison et al., 2014).

Recently, the use of ultrastructure expansion microscopy (U-ExM) in combination with immunofluorescence using Anti-BiP antibody, allowed imaging the ER of *Trypanosoma brucei*, with high resolution even under conventional light microscopy. In a nutshell, this interesting technique consists of introducing a swellable gel into fixed cells or tissues and then chemically induces its swelling, resulting in physical magnification of the specimens and increased resolution in fluorescence-based microscopy (Chen et al., 2015; Kalichava and Ochsenreiter, 2021).

## The first order ER functions in trypanosomatids

**Figure 3:**
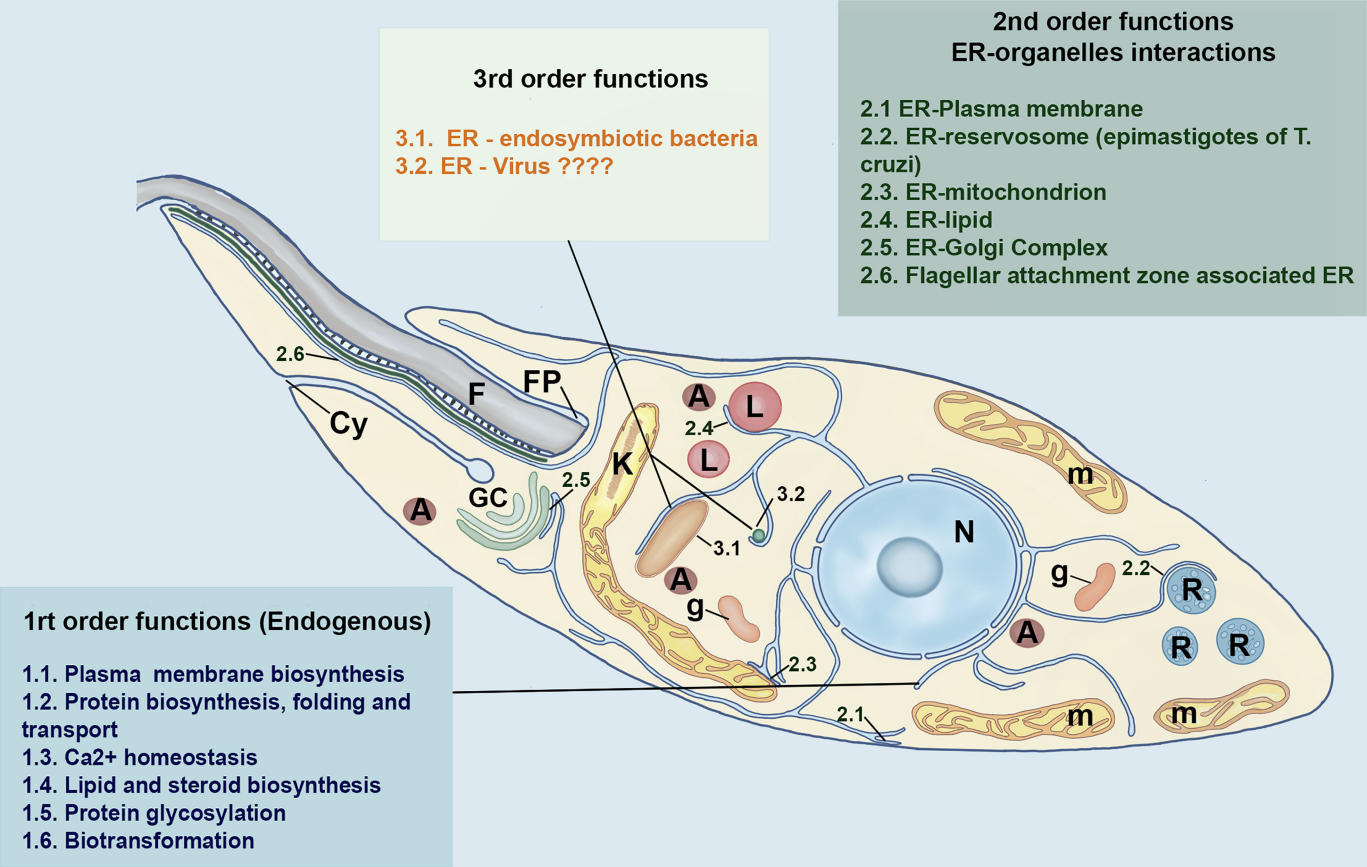
Schematic model of different functional niches of ER in Trypanosomatids based on Almeida (2021) model of a first, second and-third-order functional hierarchy for higher eukaryotic cells. The first-order functions are related to endogenous functions of the ER. The second-order is concerned with the interaction of ER with other organelles, whereas the third order includes the putative interaction of the trypanosomatid´s ER with bacteria and viruses. The main organelles of trypanosomatids are also represented. N, nucleus; A, acidocalcisomes, GC, Golgi Complex; L, lipid inclusions; m, mitochondrion; FP, Flagelar Pocket; F, Flagellum; g, Glycosomes, Cy, cytotostome and R, Reservosomes. This freehand digital illustration was made using Procreate^®^ and Adobe Photoshop^®^ apps.

From the functional perspective the ER of Trypanosomatids, like mammalian cells, may be classified in three hierarchic orders, as proposed by Almeida (2021) (**Figure 3**). The “first-order” comprises the major traditional endogenous roles of ER, i.e., the biosynthesis, folding and transport of secretory proteins, intracellular site for Ca^2+^storage, synthesis of cellular lipids and sterols, carbohydrate metabolism and biotransformation. The second-order role of the ER is regarding the way by which this organelle works as an “exchange station” establishing physical membrane contact sites (MCSs) with other organelles, notably with mitochondria, lipid droplets, and plasma membrane (Soto-Heredero et al., 2017; Almeida and Amaral, 2020; Almeida, 2021). The higher complexity of ER is not circumscribed to their endogenous functions. In this regard, in the third-order, the ER can be considered as a host contact site for endosymbiotic pathogenic and non-pathogenic bacteria, parasitic protists, and viruses. In higher eukaryotic cells, these interactions with endosymbionts might be an ancient and conserved ER mechanism, selected for communicating with mutualistic endosymbionts in specific life cycle stages, being further exploited by pathogens (Almeida, 2021).

To ensure cellular homeostasis, the first two-orders ER functions in mammalian, as well as in trypanosomatids, must be working properly, whereas the third one can be either beneficial or deleterious for the functioning of this organelle as well as the whole cell. Because of the higher complexity of ER, it is not surprising that numerous cellular mechanisms, mediated by a myriad of proteins, mainly chaperones and proteases, must exist to maintain the functional specialization and homeostasis of ER, under both physiological, and pathological conditions (Wilkinson, 2020). Molecular chaperones are specialized proteins that can interact with non-native conformation of other proteins. These proteins assume a pivotal role in the protein quality control present in the ER, ensuring that proteins are correctly folded and functional at the right place and time (Requena et al., 2015). In addition, the ER has several proteins, so-called Protein Disulfide Isomerases (PDI), that catalyses the oxidation, reduction, and isomerization of disulfide bonds of proteins. PDIs have chaperone activity facilitating the refolding of denaturated proteins that lack disulfide bridges (Padilla et al., 2003). A typical 55-kDa PDI (containing two -CGHC- active sites and four thioredoxin domains) was identified in *T. brucei* (Hsu et al., 1989; Rubotham et al, 2005) *and L. major*, but only in this latter parasite this protein was shown to possess redox/isomerase activities *in vitro*. An increased expression of PDI was found in high virulent strain of *L. major*, suggesting a role in *Leishmania* pathogenesis (Ben Achour et al., 2002). In *L. donovani* an atypical ER-localized PDI (LdPDI), containing a single thioredoxin-like domain was identified. The overexpression of the mutated or inactive form of LdPDI significantly reduced the release of secretory acid phosphatases, suggesting that this protein play a critical role in the *Leishmania* secretory pathway (Padilla et al., 2003).

Trypanosomatids exhibit divergent and intrigant secretory pathway, shaped by selective pressures to facilitate the completion of the life cycle in multiple host environments. The most striking illustration of the secretory pathway complexity in Kinetoplastids is the higher prevalence of molecules linked to glycosyl phosphatidylinositol (GPI) on the cell surface compared to the density of transmembrane proteins. In bloodstream form of *T. brucei*, for instance, the plasma membrane is decorated with 1 x10^7^ copies of the variant surface glycoproteins (VSGs) (Borges et al., 2021; de Castro Neto et al., 2021). Along contributing with the invasion process, the higher density of this class of molecule precluding immune recognition of less abundant invariant surface determinants. The insect stage, which has a less sophisticated immune system, displays 5×10^6^ copies of procyclin, a small acidic and highly glycosylated GPI-anchored protein. VSG corresponds to around 90% of total surface protein (Field and Carrington, 2004). Interestingly, the RNAi silencing of VSG causes cell cycle arrestment and altered morphology of secretory pathway, with decreased number of coupled ER exit sites and GC stacks, as well as a notable distortion of ER of these parasites. These data suggest that a reduced secretory flux of VSG, the major secretory cargo in this protozoan, impacts the morphology and maintenance of ER and Golgi structures (Sharif et al., 2022).

*T. cruzi* is also covered by a thick coat of glycoconjugates, mostly composed by a family of mucin-like glycoprotein (Acosta-Serrano et al., 2001; Borges et al., 2021). The carbohydrate in the mucins fulfils crucial functions, the most important of which being the accepting of sialic acid from the host, which is needed for infection (Giorgi and Lederkremer, 2020). In *Leishmania* spp. GPI-anchored lipophosphoglycans (LPG) and GP63 are recognized as potential virulence factors and required for transmission (Isnard et al., 2012). Consistently, a recent study showed that the loss of GPI-anchored molecules in *L. donovani*, induced by the ablation of LdPBN1, an enzyme required for the GPI-anchor biosynthesis, lead to the failure of infection in mouse model of visceral leishmaniasis (Roberts et al., 2022). The expressive predominance of GPI-anchored surface proteins indicates that the mechanisms for transport, targeting and sorting of proteins in kinetoplastids are divergent from mammalian cells, where the most surface molecules have transmembrane domains (Manna et al, 2014).

Most proteins addressed to Golgi, lysosomes, plasma membrane, and extracellular space in trypanosomatids, as well as in mammalian cells, enter the ER either during their synthesis on membrane-bound ribosomes (co-translational translocation) or after their translation has been completed (post-translational translocation). In the co-translational translocation, the protein bearing a signal peptide (SP), or transmembrane domain is recognized by signal recognition particle (SRP). The ribosome-nascent chain-SRP complex then binds to the ER membrane *via* the SRP receptor. After SRP release, the translating ribosome interacts with a complex that mediates post-translational translocation in the ER membrane, named the SEC complex translocon (Goldshmidt et al., 2008). Trypanosomes possess an unusual SRP complex that contains two RNA molecules, 7SL RNA and a tRNA-like molecule, whereas mammalian SRP is composed for a single RNA molecule (7SLRNA) and six proteins (Liu et al., 2003; Kobayashi et al., 2018). The depletion of the SRP-dependent pathway in *T. brucei* has no effect on the translocation of several SP-containing proteins, indicating that these parasites may have an SRP-independent post-translational translocation pathway. Interestingly, GPI-anchored proteins were shown to be more reliant on the SRP-independent pathway, implying that this way is the preferred route for translocation of this class of proteins. Like yeast but unlike other higher eukaryotes, trypanosomes possess the SEC complex protein SEC71, which is essential for parasite survival. This protein functions exclusively in SRP-independent translocation (Goldshmidt et al., 2008). The RNAi silencing of *SEC71* causes mislocalization of SP-proteins, reduction in GPI-anchored proteins, but did not affect membrane proteins. On the other hand, the RNAi silencing of genes encoding SRP proteins in *T. brucei* and overexpression of dominant-negative mutants of 7SLRNA in *Leptomonas collosoma* reduced the levels of polytopic membrane proteins in both trypanosomatids (Lustig et al., 2007a). In *T. cruzi* the translocation of the surface GPI-anchored GP82 trans-sialidase involves a cleavable N-terminal signal peptide followed by N-glycosylation and cleavage/addition of a GPI anchor at C-terminus. By analogy with *T. brucei*, it is possible that this protein would be transported post-translationally by an SRP-independent pathway and the SP cleaved by signal peptidase complex (Cordero et al., 2019). The silencing of *SEC63*, which encodes the SEC63 protein, required for co- and post translational translocation, prevents both polytopic proteins and GPI-anchored proteins from entering the ER, suggesting that in trypanosomes this factor is essential for both translocation routes (Goldshmidt et al., 2008). The relative promiscuity of trypanosomes in choosing a translocation route for each protein class and the existence of minimal SRP-independent apparatus can represent an evolutive gain to fast and efficiently translocate the very abundant GPI-anchor proteins (Lustig et al., 2007a).

During their journey through the ER, proteins undergo various modifications paired with chaperones to prevent misfolding, and aggregation. The first event is the cleavage of the signal peptide by a signal sequence peptidase complex followed by N-linked glycosylation, formation of intramolecular disulfide bonds and transportation from the ER to the GC for further processing (Navid and Colbert, 2017; da Costa et al, 2020). Like all the eukaryotes, most proteins addressed to the secretory pathway in trypanosomatids are modified in the ER, throughout glycosylation. For parasites as *T. brucei*, *T. cruzi* and *Leishmania* spp. glycoproteins present on the cell surface, especially those attached *via* GPI anchors, are critical for the completion of life cycle, escape from the immune defence, protection against harsh environment and interaction with hosts. In higher eukaryotes, asparagine N-linked glycosylation is an essential posttranslational modification reaction which occurs in ER and GC (Breitling and Aebi, 2013). The precursor for N-glycans is formed on the lipid carrier dolichol (Dol), found on the ER membrane. During protein translation and sequestration into the lumen of the ER, the glycan component of the final precursor Glc_3_Man_9_GlicNac_2_-PP-Dol, is transferred *en block*, by the multisubunit oligosaccharyltransferase (OST) to Asn residues within Asn-X-Ser/Thr consensus (Parodi, 1993; Jones et al., 2005). The glucose residues are further removed by two α-glucosidases (α-Glc I–II). The reverse of this reaction is performed by UGGT (UDP-glucose:glycoprotein glucosyltransferase. This protein senses the folding state of released glycoproteins and, if the correct conformation has not been achieved, UGGT reglucosylates the N-glycans to allow another cycle of calnexin/calreticulin assisted protein folding (Link et al., 2021). In *T. brucei* UGGT enzyme has an unusually wide substrate specificity, ranging from Man_5_GlcNAc_2_ to Man_9_GlcNAc_2_ glycans and are essential for resistance to higher temperatures (Izquierdo et al., 2009). The lectin (Calreticulin)-N-glycan-mediated quality control of glycoprotein folding in *T. cruzi*, is exclusively formed by UGGT-dependent glycosylation. In *T. cruzi*, for example, about 65% of glycans in either the C-terminal extension or in the catalytic domains of cruzipain, a lysosomal proteinase and important virulence factor in this protozoan, are glycosylated by TcUGGT allowing it to interact with calreticulin (CRT). Surprisingly, Conte et al. (2003) demonstrated that disruption of both Tc*UGGT* alleles only partially decrease the cellular content of CRT (5-20%) and, as expected, no CRT-cruzipain interaction was identified. In this case, CRT interacts with GRP78/BiP (binding-immunoglobulin protein aka GRP-78) for significantly longer time periods, indicating that an alternative folding facilitation mechanism, independent of CRT, is triggered to avoid the catastrophic consequences of TcUGGT absence (Conte et al., 2003).

Because trypanosomatids were deficient in the formation of dolichol-P-Gly, they are the only eukaryotic cells known so far, that transfer *in vivo* unglucosylated oligosaccharides during N-glycosylation of proteins. Furthermore, some species lack certain dolichol-P-Man-dependent mannosyltrasnferases. The composition of these oligosaccharides varies with the species and life stage of the trypanosomatid (Parodi, 1993). In *T. cruzi*, for example, only (Man_5_-Man_9_)-GlcNAc_2_-PP-Dol were observed for epimastigotes, whereas trypomastigotes displayed a larger repertoire of more elongated (i.e., Man 10) and truncate (man-Man_4_) mannose glycans (Engel and Parodi, 1985; Alves et al., 2017). *Crithidia fasciculata* possess Man_7_GlcNac_2_-PP-Dol (Parodi et al, 1981). In promastigotes of *Leishmania spp* the oligosaccharide Man_6_GlcNAc_2_-PP-Dol is consistently found, whereas in amastigotes, alterations in N-linked oligosaccharides appeared to be less consistent between species (Zamze, 1991). *L. mexicana* amastigotes were found to display the same N-modified glycans whereas *L. donovani* amastigotes were found to be devoid of N-linked glycans (Funk et al, 1997).

The terminal mannose in trypanosomatids (and glycose in mammals) are essential to the correct folding of newly glycosylated proteins and to ensure glycoprotein quality in the ER (Mule et al., 2020). Another divergent characteristic of N-glycosylation in these parasites is that dolichol chains are shorter (10-13 isoprene units) than in mammals (18-21 isoprene units) and fungi (15-16 isoprene units). Furthermore, glucosidase II-like enzyme is present in trypanosomatids, but glucosidase I is lacking (Parodi, 1993). The *en block* transfer of a glycan from a lipid-linked oligosaccharide (LLO) donor to a nascent polypeptide chains acceptor is also catalysed by OST enzyme in ER. Kinetoplastids encode a single catalytic subunit of OST enzyme, STT3, whereas, depend on the species, higher eukaryotes encode OST with different complexity (Poljak et al., 2017; Mule et al., 2020). *T. brucei* encodes three paralogue of single-protein OSTs called TbSTT3A, TbSTT3B, and TbSTT3C, that can functionally complement the *Saccharomyces cerevisiae* OST. These STT3s display distinct preferences for LLO donor as well as for the acceptor polypeptide substrate (Jinnelov et al., 2017; Poljak et al, 2017).

The Ca^2+^ content in ER is critically important for maintaining its oxidizing environment as well as the luminal ATP levels required for chaperone activity. As a result, both the luminal Ca^2+^ concentrations and the dynamic Ca^2+^ flux between the different subcellular compartments should be tightly controlled (Daverkausen-Fischer and Pröls, 2022). Ca^2+^signalling pathways in trypanosomatids are highly divergent from those in the mammalian hosts and other higher eukaryotes, which can be promissory target for therapeutics (Benaim et al., 2020). Firstly, whereas mammalian cells use the endoplasmic reticulum as a main Ca^2+^ storage site, trypanosomatids store most of their Ca^2+^ in an acidic organelle named the acidocalcisome (Docampo and Huang, 2022). Accordingly, the Ca^+2^ export channel, inositol 1,4,5, triphosphate receptor, which localizes to the ER in mammals, in trypanosomatids it localizes to acidocalcisome (Huang et al., 2013; Ramakrishnan and Docampo, 2018). The uptake of Ca^+2^ in mammalian cells is mediated by the sarco/endoplasmic reticulum Ca^2+^ calcium ATPase (SERCA). Orthologs for the SERCA-type Ca^2+^ ATPase protein has been identified and characterized in *T. brucei* (Nolan et al., 1994), *T. evansi* (Mendoza et al., 2004; Pérez-Gordones et al, 2015), *T. cruzi* (Furuya et al, 2001) and *Leishmania spp* (Lu et al., 1997; Rodriguez et al, 2002, for review see Meade, 2019). In *T. brucei*, this Ca^2+^ ATPase has high affinity for Ca^2+^ and is sensible to vanadate and to low concentrations of thapsigargin (TG), a long known specific SERCA inhibitor (Nolan et al., 1994). However, *T. cruzi* showed to be resistant to low concentrations of TG. The differences in the susceptibility to TG have been attributed to distinct transmembrane sequences in the SERCA TG-binding site in these parasites (Furuya et al, 2001). Molecular modelling indicates that TG or its analogues might be used as a potential drug against trypanosomes (Pérez-Gordones et al, 2015). In *L. amazonensis*, a Ca2+-ATPase LmAA1 is located in the ER and act as a virulence factor, i.e., strains overexpressing LmMA1were more infective in both mouse and *in vitro* macrophage infection experiments (Rodriguez et al, 2002). The transfection with LmAA1 also lead to improved survival of stationary-promastigotes suggesting that the primary role of this proteins may be in metacyclogenesis (Rodriguez et al, 2002).

Whereas SERCA is the primary calcium transporter/PUMP, the chaperone calreticulin (CRT) is important for ER calcium buffering in trypanosomes, since calnexin, another chaperone involved in this process, is absent in these protozoans. CRT is also a central component of the glycoprotein folding quality control in the ER. CRT is formed by three structural domains: (i) the N-terminal domain contains the sugar binding site; (ii) the proline rich or P-domains which participates in glycoprotein binding by interacting with Erp57; and (iii) the C-terminal domain, which is rich in negatively charged residues responsible for the calcium buffering activity (Labriola et al., 2010). Repeated glycosylation and deglycosylation ensure that misfolded glycoproteins spend enough time in the ER to be folded correctly (Chakrabarti et al, 2011; Hetz, 2012).

The CRT of *T. cruzi* (TcCRT) shows similar structural and functional characteristics found in CRT of other eukaryotic cells. TcCRT is majority an ER-resident protein binding to monoglusylated glycans. This protein is also involved in the retention of immature proteins in the ER. In particular TcCRT plays an important role in the maturation of cruzipain (Conte et al., 2003). Although TcCRT is located mainly in the ER, histochemical studies have found it in the Golgi complex, reservosomes, flagellar pocket, cell surface cytosol, nucleus and kinetoplast. These alternative locations are probably linked to the diverse biological roles played by this diverse chaperone. The mechanism behind the localization of CRT beyond ER sites is not completely known. However, it has been postulated that the conformation of CRT-C terminal domain is affected by calcium level. ER calcium depletion (but not by the increase in cytosolic calcium), triggers the retrotranslocation of TcCRT in a relatively short period of time, resulting in its clearance from cytoplasm *via* the proteasome (Labriola et al., 2010). The TcCRT, translocated from the ER, is addressed to plasma membrane and released to extracellular medium, where it acts as a complement inhibitor and a virulence factor. TcCRT protein may be also involved in an antitumor effect by acting as a potent angiogenesis inhibitor (Molina et al., 2005).

## The second-order role of ER in trypanosomatids

Given its dynamic nature and critical roles in producing proteins and lipids for other organelles and preserving Ca^+2^ equilibrium, is reasonable to assume that a sophisticated communication system must exist between ER and other organelles and endomembranous compartments. The ER communication with other organelles can be achieved by vesicular traffic or by membrane contact sites (MCSs). The MCSs are regions where two organelles are about 30 nm apart from one another and their membranes are linked by protein-protein and protein-lipid interactions, which aid to maintain membrane closeness while avoiding membrane fusion (Santos and Nozaki, 2021). Although MCSs between the ER and other organelles such as the plasma membrane (PimentaDe Souza, 1985), lipid droplets (Soares and de Souza, 1987; Pereira et al., 2018), reservosomes (epimastigote of *T. cruzi*) (Figueiredo et al., 2004) (**Figure 2A**), and particularly the mitochondrion (Pimenta and De Souza, 1985) have long been observed in trypanosomatids by TEM, our knowledge of their functions and organization has only recently improved as a result of advances in electron and light microscopy resolution and the development of novel unique fluorophores. (Wu et al., 2018).

In mammalian cells ER-plasma membrane (PM) contacts mediate numerous inter-cellular and intra-cellular signalling pathways including phosphoinositide lipid and calcium signalling, mechanotransduction, metabolic regulation, and cell stress response (Stefan, 2020). The close proximity of ER and PM observed in trypanosomes ( **Figure 2**), strongly suggests that ER-PM contacts also play a role in membrane lipid and ion dynamics in these parasites. Consistently, an extended synaptotagmin (E-Syts), belong to a family of evolutionarily conserved proteins localizing at the ER-PM MCS, was described in *T. brucei* (TbE-Syt). In mammalian cells E-Syts are Ca^2+^-dependent lipid transfer proteins at the membrane contact sites (Yu et al., 2016; SahekiDe Camilli, 2017). Like to all mammalian cells, E-Syts from *T. brucei* has an N-terminal transmembrane hairpin and a central synaptotagmin-like mitochondrial lipid binding protein (SMP). However, TbE-Syt contains only two C2 domains, named C2A and C2B in contrast to at least three C2 domains in all other reported E-Syts present in higher eukaryotic cells (SahekiDe Camilli, 2017; Stepinac et al., 2021). The depletion of TbE-Syt had no significant effect in bloodstream form *T. brucei* but have deleterious effect in procyclic forms. Immunocytochemistry analysis showed that TbE-Sys co-localizes to both BiP, at the central ER close to nuclear envelope, and (FAZ1) a marker protein of unique flagellar attachment zone (FAZ) – associated cortical ER (Stepinac et al., 2021). The FAZ is a large macromolecular structure located at the interface formed by the cellular and the flagellar membranes. It is composed of a filament spanning the cellular and flagellar membranes, a set of four microtubules, called the microtubule quartet, and a FAZ-associated endoplasmic reticulum (Trépout, 2020). The analysis of 1.5-Å-resolution crystal structure and the homology modelling of TbE-Syt-C2B revealed that it binds lipids *via* both Ca^2+^ and PI(4,5)P2 dependent manner. The TbE-Syt might thus anchor and staple the FAZ-associated cortical ER to the plasma membrane, allowing the dimeric SMP domains, to transport lipid molecules in their hydrophobic clefts, from ER to the plasma membrane. (Stepinac et al., 2021).

The best-characterized MCSs in mammalian cells are established between the ER and mitochondria (ER mitochondrial-associated membranes, MAMs). These contacts are critical for many intracellular processes, and a defect in MAMs or their homeostasis, has been associated with certain pathological conditions (Soto-Heredero et al, 2017). The ER membrane protein complex (EMC) has recently emerged as an important eukaryotic complex for biogenesis of integral membrane proteins by promoting insertion and stability of atypical and sub-optimal transmembrane domains. Primarily, the EMC, a multimeric insertase consisting of 8 (yeast) or 9 (animals) subunits, is involved in the topogenesis of multispanning ER proteins, either independently or in conjunction with the SEC61 translocon. However, their roles as an ER-contact site and an inter-organellar lipid exchange site in mammalian and trypanosomatids have recently been described. (Volkmar and Christianson, 2020). In a study by Iyer et al. (2022), biochemical, proteomic, and imaging approaches were used to study the EMC (TbEMC) in *T. brucei*. This study revealed that TbEMC is formed by 9 subunits with 20-40% of similarity with yeast and human homologs. These proteins are localizing to the mitochondrial-ER interface, suggesting that certain subunits can be involved in the transfer of phospholipid precursors or intermediates between these organelles. Indeed, the knocking out or knocking down of single TbEMC subunits led to growth defects on *T. brucei* procyclic forms and the disruption of *de novo* synthesis of phosphatidylcholine and or phosphatidylethanolamine, the two most abundant phospholipid classes in *T. brucei*, pointing to a role of the TbEMC in phospholipid synthesis. In addition, in TbEMC7 knockout parasites, TbEMC3 is released from the complex, implying that TbEMC7 is essential for the maintenance of the TbEMC integrity (Iyer et al., 2022).

## The third order function of ER: Living with the others

The presence of intracellular viruses and bacteria in trypanosomatids has long been recognized. However, data on the influence of these microorganismis on trypanosomatids’ biology and their relationship with ER is limited, and most studies were based solely on morphological observations by TEM (Cantanhêde et al., 2021). Six species of Trypanosomatids found in insects harbour a single obligate intracellular bacterium in their cytoplasm, with *Angomonas deanei* and *Strigomonas culicis* (formerly named as *Crithidia deanei* and *Blastocrithidia culicis*, respectively) being the best characterized species by ultrastructural and biochemical approaches (Motta et al., 2013). Trypanosomatids have also been reported to be infected by a wide range of unrelated RNA viruses. (Grybchuk et al, 2018). In *Leishmania* spp., infection with RNA viruses causes a stressful condition, promoting increased tolerance of these parasites to some environments conditions and augmenting their replication rate, thus influencing the leishmaniasis pathogenesis (Ives et al., 2011; Grybchuk et al, 2018). Because viruses are obligate intracellular parasites that subvert the function of their host to replicate and form new viral particles, it is not surprising that ER has been identified as a central organelle that governs the intracellular interplay between viruses and hosts throughout the entire viral life cycle. Therefore, the ER may be not only essential in maintaining cellular homeostasis but also may render cells particularly susceptible to viral infection, no matter whether it is a higher or lower eukaryotic cell. Consistently, Fernández-Presas et al. (2017) reported the presence of both enveloped and non-enveloped virus-like particles (VLPs), with 48 and 32 nm size, respectively, in the cytoplasm of *T. cruzi* epimastigote forms. Clusters of electrodense enveloped, as well as a paracrystalline array of non-enveloped VLPS, were found in distended Golgi cisternae or as smaller agglomerates near to the RE or Golgi (Fernández-Presas et al., 2017).

The ER is also useful for the establishment of relationships with intracellular pathogenic or non-pathogenic bacteria. The protozoan *Angomonas deanei*, a trypanosomatid parasite of the gastrointestinal tract of insects, harbours a single endosymbiont in its cytoplasm. Recently, an elegant study by Catta-Preta and colleagues (2022), using FIB-SEM and proteomic analysis, showed a close association of symbiont bacteria with the ER, nucleus and, glycosomes of *A. deanei*. Multiple ER connections were found in dividing cells, mainly in the area of bacterial membrane constriction. Interestingly, the authors stated that no membrane fusion between the endosymbiont envelope and the parasite reticulum was observed, suggesting that MCS between the endosymbiont and ER may exist. Sometimes this association is so close that the symbiont is seen to be fully wrapped by the ER (Catta-Preta et al., 2022). Tunicamycin, a naturally occurring antibiotic that inhibits the earliest steps of N-glycosylation, interrupts this mutualistic interaction by increasing the distance between the symbiont and ER. These findings support the hypothesis that the optimized functionality of the ER in *A. deanei* is important for maintaining the mutualistic relationship between the bacterium and the parasite (Catta-Preta et al., 2022)

## ER-quality control and ER-response to stress in trypanosomatids

In higher eukaryotes, more than a third of the proteome translocates across the ER membrane and enters the endoplasmic reticulum (ER) during or soon after synthesis (Kumari and Brodsky, 2021). As mentioned above, protein folding within the ER is mediated by chaperones, PDIs, and cycles of glucosylation and deglucosylation, which lead to successful completion and exportation to the GC or, instead, its retrotranslocation into the cytosol. Rejection of misfolded polypeptides requires mannosidase II action to produce Man_8_GlcNAc_2_ oligosaccharide, which is recognized by ER-Degradation Enhancing α-mannosidase (EDEM)- like protein (Field et al., 2010). The constant influx of newly synthesized proteins must be strictly controlled to prevent their production exceeding the ER’s processing capacity (Sun et al., 2021), which can cause an accumulation of misfolded/unfolded proteins and ER stress. Because misfolded/unfolded proteins can induce aggregation, which is harmful to the cells, they are not tolerated and are usually eliminated by two efficient control/quality systems: the Endoplasmic Reticulum-Associated Degradation (ERAD) and the reticulophagy (ER-phagy). The ERAD is the main degradation pathway for soluble proteins, whereas the ER-phagy allows the degradation of part of ER, membrane lipids, and non-soluble aggregates (Navid and Colbert, 2017; Chino and Muzushima, 2020).

The ERAD is a multi-step quality control mechanism that requires a sophisticated set of components to remove aberrant proteins that threaten the ER and hence, the cellular equilibrium (Kumari and Brodsky, 2021). During ERAD, the aberrant protein is first recognized by chaperones, and after its selection, it is retrotranslocated back to the cytosol to be ubiquitinated. First, the ubiquitin-activating enzyme (E1) hydrolyses ATP and forms a high-energy thioester linkage between its active site cysteine and the carboxy terminus of ubiquitin (Ub). Activated ubiquitin is then transferred to a member of the family of ubiquitin-conjugating enzymes (Ubc or E2). E2 enzymes, together with ubiquitin protein ligases (E3) preferentially attach ubiquitin to Lys residues of substrate proteins, but other residues, such as threonine, serine, and cysteine, and the N-terminal amino group can be modified as well (Bijlmakers, 2021).

Although it has not yet been extensively investigated in kinetoplastids, data from genome-wide RNAi and the use of pharmacological inhibitors of trypanosome 26S proteasome have demonstrated the presence of an ERQC system in trypanosomes. Consistently, genes encoding for orthologues of ubiquitin E1, E2 and E3 proteins are also present in these parasites (Field et al, 2010; Gupta et al., 2018; Bijlmakers, 2021). In higher eukaryotes, UBA1 is the E1 ubiquitin protein responsible for more than 90% of all ubiquitination processes. In *T. brucei*, there are two orthologues of UBA1, called TbUBA1a and TbUBA1b. As expected, Tb*UBA*1b RNAi knockdown results in an overall reduction in ubiquitination in the bloodstream forms of the parasite, and both TbUBA1a and TbUBA1b are essential for its survival (Boer and Bijlmakers, 2019). The polyubiquitin chains are the principal signal for addressing ERAD subtracts to the 26S proteasome and a minimum of four ubiquitins are required for proteasome-dependent degradation (Meusser et al., 2005; Goder, 2012). The ubiquitinated protein is recognized by cytoplasmic p97 (aka VCP or Cdc48) and extracted from the ER membrane using energy from ATP hydrolysis (Garrison and Bangs, 2020). A functioning ERAD pathway has been found in *T. brucei* (Tiengwe et al., 2018; Garrison and Bangs, 2020), *T. cruzi* (Labriola et al., 2010; Gupta et al., 2018) and *Leishmania*, and several orthologues of all key components of the ERAD pathway are also present in these parasites (Gupta et al., 2018). Differently from yeast and mammalian cells, ERAD is suggested as the main mode of disposal of misfolded GPI-anchored proteins in *T. brucei* (Garrison and Bangs, 2020; Bijlmakers, 2021). Because of its essential function, the ubiquitin-proteasome system represents a good potential drug target in trypansomatids (Harbut et al., 2012). In this regard, the potential of TAK-243 a potent inhibitor of human UBA1, has been tested against *T. brucei*. Interestingly, both UBA1a and UBA1b were shown to be resistant to these inhibitors, demonstrating that TAK-243 binding sites in humans and in *T. brucei* differ significantly (Boer and Bijlmakers, 2019; Bijlmakers, 2021). Thus, using TAK-243 as a starting point, novel molecules that bind to TbUBAs but not huUBAs may be developed, based on the differences at the TAK-243 binding-pocket (Boer and Bijlmakers, 2019; Bijlmakers, 2021).

Although the ERAD efficiently detects, selects, and degrades misfolded and/or abnormal proteins *via* proteasomes, the size restriction of the retrotranslocation pore may prevent certain proteins and aggregates from being addressed to the ERAD system. Thus, the autophagic degradation of the ER, also known as ER-phagy or reticulophagy, is the second arm of the ER-based degradation and quality-control (Chino and Muzushima, 2020; Molinari, 2021). In general, ER-phagy serves to assist the autophagic pathway towards the fast mobilization of nutrients during cell starvation; constitutively regulating the size and shape of ER and eliminating aberrant proteins and aggregates (Reggiori and Molinari, 2022).

The autophagic process in trypanosomatids has been long recognized by the observation of routine TEM processing samples submitted to stress conditions that often trigger this process in higher eukaryotes, such as nutrient deprivation, pharmacologic treatment, and ER-induced stress. For example, in our previous work, the treatment of *T. cruzi* epimastigotes with tunicamycin (TM) caused an increase in ER profiles surrounding cytoplasmic organelles or even bulk cytoplasmic portions. The appearance of myelin-like structures, increased numbers of larger autophagosomes and multivesicular bodies, characterized by the presence of small vesicles inside great lysosomal compartments, were also usually observed in TM-treated parasites (**Figures 2D–E**). These results suggested that the ER-phagy process was taking place (Sandes et al., 2019). However, in this study, it was not possible to distinguish the specific ER-phagy from the global autophagic process except by the fact that the ER-stressor was used. The autophagy is highly conserved among eukaryotes (for review see Pedra-Rezende et al., 2022) and many autophagy-related gene (ATG) homologs have been identified in trypanosomatids, including the complete conjugation system (*ATG3*, *ATG4, ATG7*, and *ATG 8*), involved in phagophore elongation and degradation of autophagosome cargo (Li et al, 2012; Pedra-Rezende et al., 2022).

## The ER-response to stress: A delicate equilibrium between life and death

In addition to increased protein influx and synthesis under physiological conditions, a variety of factors, including the calcium imbalance, nutrient deprivation, deficiencies in post-translational modifications, inefficiencies in degradation pathways such as ERAD and autophagy, can trigger ER stress (Bagchi et al, 2021). Compounds that prevent the first steps of N-glycosylation in the ER (tunicamycin, TM) or disulfide bond formation (dithiothreitol, DTT), disrupt chaperone function (thapsigargin, TG), or induce retrograde transport of protein from the Golgi to the ER (brefeldin A, BFA) are also potent inducers of ER stress in eukaryotic cells (Navid and Colbert, 2017).

**Figure 4:**
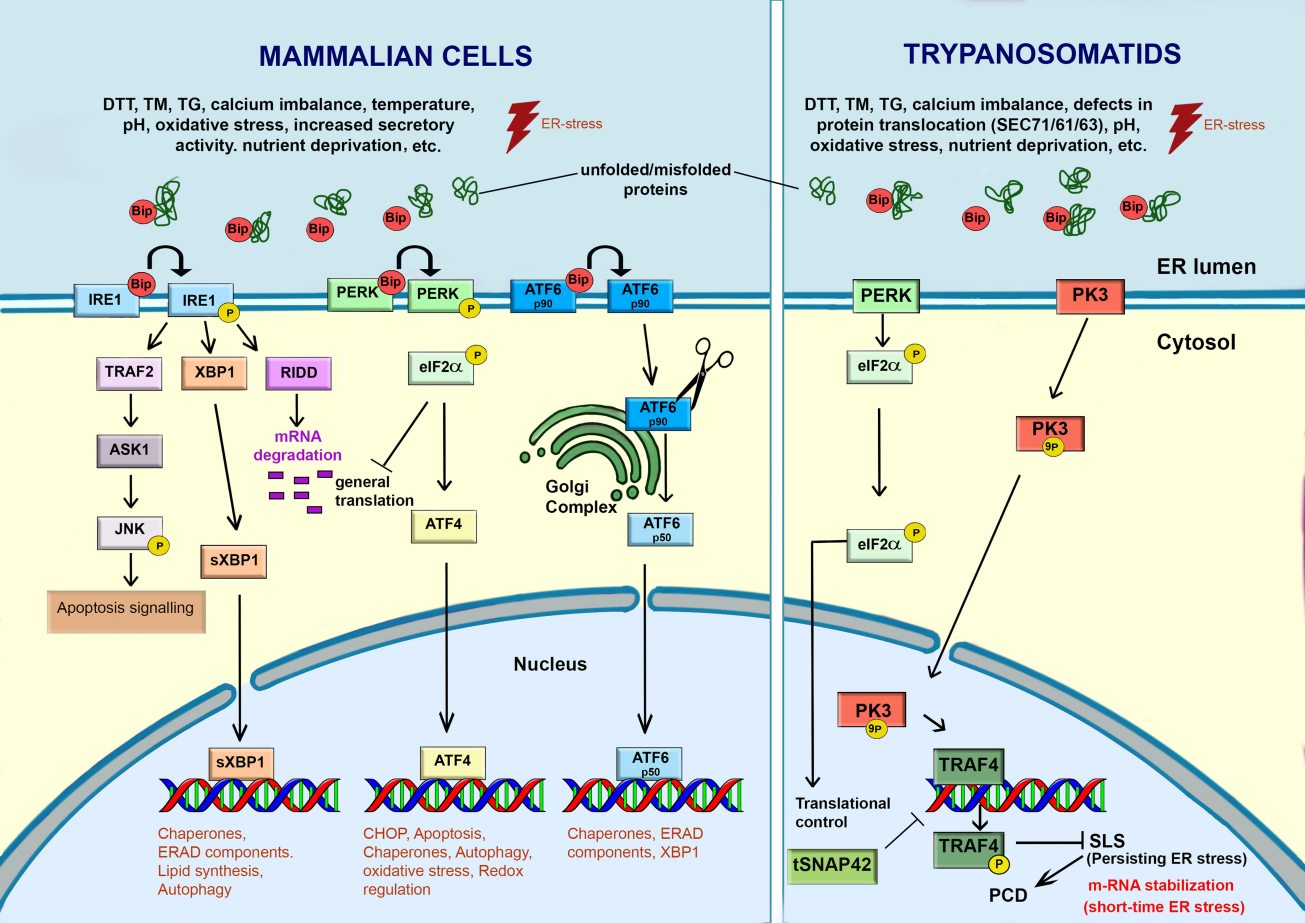
Response to ER-stress in mammalian cells and trypanosomatids. In mammalian cells, the accumulation of unfolded or misfolded proteins, induced by various intrinsic and extrinsic stimulus, causes ER stress. Under stress, BiP/GRP78 dissociates from the ER transmembrane stress sensors (IRE1, PERK and ATF6) and activates a signaling cascade to re-establish the ER functions and promote cell survival through the transcription of genes related to autophagy, chaperones, lipid biosynthesis, ERAD, etc. However, failure of this adaptative UPR response may activate programmed cell death (PCD). Trypanosomatids lack a canonical UPR response. IRE1 and ATF6 are absent in these parasites. A PERK homologue was found in *Leishmania* spp. and *T. cruzi*. In *Leishmania* spp., ER-stressors activate PERK, which in turn phosphorylates eIF2α. The phosphorylation of eIF2α leads to attenuation of translation and a decrease in the load of proteins into the ER. In *Trypanosoma brucei*, PK3, a serine threonine kinase, plays a key role in sensing and transmitting the ER-stress signal from ER to the nucleus, where it activates the Splice Leader Silencing (SLS) pathway. PK3 phosphorylates TRF4, causing its release from its cognate promoter. The hallmarks of SLS are the shut-off in SL RNA due to impairment of tSNAP42’s ability to bind the SL RNA promoter, leading to its accumulation in the nucleus. Under mild ER-stress, some mRNAs are stabilized. When the ER-stress is persistent, the PK3-TRAF4-SLS cascade leads to the cell death. This freehand digital illustration was made using Procreate^®^ and Adobe Photoshop^®^ apps.

Upon ER stress, cells activate a series of complementary adaptative responses to cope with the accumulation of unfolded/misfolded proteins into the ER, collectively known as the unfold protein response (UPR) (Hetz, 2012) ( **Figure 4**). Regardless of the stressor stimulus, the primary function of UPR is to re-establish ER homeostasis by reducing protein synthesis, increasing protein folding, and accelerating misfolded protein degradation (Yang et al., 2021). This appears to be the scenario reported in an early work on the effects of TM on *Leishmania brasiliensis* (Dagger et al., 1984). In this study, long-term exposure of promastigotes to this ER stressor, resulted in moderate growth inhibition, cell rounding, and the formation of numerous ruffle-like structures at the cell surface, followed by the loss of cell coat due to the decrease in total protein synthesis. Although the mechanisms underlying promastigote tolerance to TM were unknown at the time, these findings demonstrated the presence of an adaptative response to TM injury.

In mammalian cells (**Figure 4**), UPR is activated by three unique transmembrane ER stress sensors: pancreatic ER kinase-like ER kinase (PERK), inositol-requiring enzyme 1 (IRE1), and activating transcription factor 6 (ATF6) (Adams et al., 2019). Under physiological conditions, PERK, IRE1, and ATF6 are bound to the chaperone BiP/GRP78 in the ER lumen. The primary function of BiP is to bind to nascent polypeptide chains to prevent their aggregation and subsequently assist them to achieve their native conformation (Adams et al., 2019; Yang et al., 2021). BiP orthologues were reported in trypanosomatids. In *T. cruzi*, this protein is an ER resident chaperone sensitive to ER-stress stimulus by TM, but resistant to heat shock (Tibbetts et al, 1994). Upon ER stress, the proteins (PERK, IRE1, and ATF6) dissociate from BiP, exerting their biological effects on downstream molecules to relieve the stress (Yang et al, 2021). Thus, the most common way to access UPR activation is evaluating the upregulation of BiP and other chaperones as protein disulfide isomerase (PDI) (Tiengwe et al., 2015).

Each one branch of UPR works in an integrated way to re-establish the ER functionality. The dissociation of IRE1 from BiP lead to autophosphorylation of IRE1-kinase domains, activating its endoribonuclease function that cleaves the transcription factor mRNA *XBP1*. After processing of the mRNA and its translation, this factor activates the transcription of UPR target genes, involved in increased chaperone folding production, lipid synthesis, expansion of ER size and synthesis of ERAD components. Furthermore, IRE1 also activates IRE1-dependent decay to decrease m-RNA (RIDD) translation and alleviate the load on protein folding machinery. However, if all these measures fail, persistent stress can lead IRE1 to interact with the TNF-receptor-associated factor 2 (TRAF2) and the apoptosis signal-regulating kinase (ASK-1) apoptosis, phosphorylating c-Jun N-terminal kinase (JNK). JNK phosphorylation results in the stimulation of pro-apoptotic factors BID and BiM, whilst inhibiting anti-apoptotic factors BCL-2, BCL-XL, and MCL-1 (Chen and Brandizzi, 2013; Adams et al, 2019). The activation of PERK, through a mechanism involving dimerization and autophosphorylation, leads to the phosphorylation of the eukaryotic translation initiation factor 2α (eIF2α) resulting in a decrease of protein loading in the ER, alleviating the ER stress. PERK also allows the selective translation of mRNA-encoding the transcription factor ATF4, which is translocated to the nucleus, where it upregulates the expression of ER chaperone (GRP78/BiP and GRP94), genes involved in ER-phagy, amino acid biosynthesis, protein secretion and antioxidant response. During long-term ER stress, ATF4 stimulates genes of CCAAT-enhancer-binding protein homologous protein (CHOP), which is responsible for initiation of the apoptotic cascade (Rozpedek et al., 2016). In this regard, depending on the intensity and duration of ER stress, the UPR response may promote pro-survival process, or in case of persistent insulting, lead cell to death by apoptosis (Kabir et al., 2018).

The trypanosome proteome contains homologs of eukaryotic initiation factor 2 (eIF2) kinases, which play a role in the UPR in other organisms. However, in *T. brucei*, a homolog of eIF2α kinase (TbeIF2K2) has a transmembrane domain but is unlike to act as UPR sensor because it localizes to the flagellar pocket where it is functioning in endocytosis (Moraes et al., 2007). Furthermore, TbeIF2K2 did not respond to ER-stress. On the other hand, a homolog of PERK was identified in the ER of *Leishmania* spp., which functions in the differentiation process from promastigote to amastigote, being hyperphosphorylated under ER-stress in this parasite (Chow et al., 2011). The *T. cruzi* PERK homolog (TcK2) is also required for the differentiation of epimastigote to infective metacyclic form and is activated by starvation but localizes in endosomes instead the ER (Tonelli et al., 2011; da Silva Augusto et al, 2015). Unlike IRE1α and PERK pathways, the release of BiP from the ER luminal domain of ATF6α triggers its transport to the Golgi *via* COPII-containing vesicles. In the Golgi, the cytoplasmic domain of ATF6α is phosphorylated and cleaved. The released fragment acts as a transcription factor that is translocated to the nucleus activating target genes whose products contribute to protein folding and secretion, ER expansion and ERAD.

Trypanosomatids, like other protozoans, diverged very early from the eukaryotic lineage. As these parasites do not control gene expression at the transcriptional level, they respond poorly to internal or external perturbation, such as heat stress or drug toxicity. The genes in these protozoans are transcribed into polycistronic primary transcripts, and the synthesis of functional mRNA is reliant on: (i) a trans-splicing reaction, in which a 5´capped 39 nucleotide spliced leader (SL) is transferred from an SL donor RNA (SL RNA) to the 5´end of each nascent mRNA; and (ii) polyadenylation of the upstream gene (Michaeli, 2011; Michaeli, 2012; Michaeli, 2015). No promoters upstream to protein coding genes transcribed by polymerase II were identified. Consequently, expression levels are primarily controlled by mRNA stability (Tiengwe et al., 2015). Because UPR activation in higher eukaryotes is performed by increased transcription of specific mRNA, it is unlikely that trypanosomatids have a classical UPR machinery (Hope et al., 2014; Tiengwe et al., 2015; Okalang et al., 2021). However, trypanosomatids are able to respond ER stress by upregulating BiP, triggering the splice leader silencing (SLS) that in turn, inhibits SL RNA transcription. The RNAi of the SRP receptor (SRα) in procyclic stage of *T. brucei*, where the ER stress is most studied, provided the first evidence of such a response. This caused the accumulation of SRP on ribosomes and triggered silencing of SL RNA by preventing the SL RNA-specific transcription factor tSNAP42 to bind to its nuclear promoter. The hallmark of SLS is therefore the expansion of ER, the shut-off of SL RNA transcription, followed by massive accumulation of tSNAP42 in the nucleus, thus stopping parasite´s ability to produce mRNA (Goldshmidt et al, 2010); Lustig et al., 2007b). Further studies found that prolongated ER stress stimulus induced by chemicals, RNAi silencing of *SEC63*, or *SEC61*(the translocation channel) lead to SLS and to cell death in both bloodstream and procyclic forms of *T.brucei*. Furthermore, treatment of procyclic forms with DTT lead to an upregulated expression of genes involved in protein folding and other key steps of canonical UPR in mammalian cells (Goldshmidt et al., 2010). This data indicates that in the absence of canonical UPR, trypanosomatids respond to ER short-term and mild stress by stabilizing specific mRNAs, which is essential for alleviating the ER stress and recovering the organelle homeostasis. However, under prolonged ER stress the SLS is triggered (Goldshmidt et al, 2010; Goldshmidt and Michaeli, 2011). As a result, these authors hypothesized that SLS is used to accelerate the death process elicited by ER stress, hence providing a mechanism to eliminate unsuitable parasites from the population (Michaeli, 2012). Indeed, typical hallmarks of PCD such as phosphatidylserine (PS) exposure, increase of cytoplasmic Ca2+, depolarization of the mitochondrial membrane potential (ΔΨm) and ROS production were observed in treated cells. Some of these apoptosis features were also found in bloodstream forms of this parasites, under ER-stress induced by DTT.

The delicate balance between life and death decisions under ER stress, as well as the difficulties in delineating its borders in trypanosomatids, may lead to some divergences of whether or not UPR-like response is present in trypanosomatids. For example, contrary to the findings of Goldshmidt et al (2010), a prior study on the *T. brucei* transcriptome under ER-stress found only minor alterations in response to both DTT (1 mM DTT, 1-4 hours) and TM (5 µg/mL, 1-24 h) treatments (Koumandou et al., 2008). Furthermore, this study also failed to detect changes in Bip expression, leading the authors to conclude that UPR-like response might not exist in trypanosomes (Michaeli, 2011). Tiengwe et al. (2015) showed that UPR-like and SLS responses did not occur in bloodstream forms of *T. brucei* submitted to the treatment with both DTT, as well as under silencing of core translocon Tb*Sec61*. The treatment with TM and TG at concentrations compatible with short-term and long-term pharmacological treatment, likewise, failed to induce any of UPR-like or SLS indicators. Furthermore, these authors argued that DTT was lethal to parasites by causing a strong redox stress, at all concentrations tested, making it unsuitable for use as an ER-stressor (Tiengwe et al, 2015; Sandes et al., 2019). The discrepancy in the phenotypes found following DTT treatment may be attributed to differences in the drug concentrations used in these studies as well as the distinct susceptibility existing between the evolutive forms of this parasite (Goldshmidt et al., 2010; Goldshmidt and Michaeli, 2011; Okalang et al, 2021). In *A. deanei* TM treatment at higher concentrations caused a decrease in proliferation at higher concentrations. Under this condition ultrastructural alterations as the enlargement of ER and Golgi apparatus cisternae were also observed (Catta-Preta et al, 2022).

Regardless the above-mentioned controversies, the mechanism underlying the ER-stress induced SLS began to be unravelled by Hope et al. (2014). These authors demonstrated that a putative PERK paralog eIF2K3, hereinafter referred to as PK3 plays an essential role in the activation of SLS pathway. The ER-stress induced by *SEC63* silencing or low pH results in the translocation of PK3 from the ER to the nucleus where it phosphorylates the TATA-binding TRF4, causing the shutoff of SL RNA transcription, followed by induction of programmed cell death. The loss of PK3 function attenuates the programmed cell death triggered by ER stress, indicating that SLS may contribute to programmed cell death activation in *T. brucei* (Bindereif and Preußer, 2014; Hope et al., 2014; Okalang et al, 2021).

In *Leishmania major* the ER stress response seems to be related to UPR as demonstrated by Dolai et al. (2011). The treatment with TM induced an increase in BiP expression and PCD phenotypes characteristic of apoptosis. Some of these effects were abolished by the pre-treatment with antioxidants, chemical chaperone (4-phenylbutyric acid) or Ca^2+^ chelators, but not with the caspase inhibitor benzyloxycarbonyl-VAD-fluoromethyl ketone or the metacaspase inhibitor antipain, suggesting that the mechanism that trigger apoptosis in trypansomatids is highly divergent from those found in higher eukaryotes (Smirlis et al., 2010). Moreover, Gosline and colleagues (2011) showed that the ER stress response of *L. donovani* may be different from *L. major*, as the first ones showed no change in BiP protein levels when treated with TM (50 µg/mL) or DTT (1mM). The DTT-treated-cells also exhibited increased eIF2α phosphorylation, suggesting that the PERK-eIF2a associated translational attenuation pathway would play a role on ER-stress response (Gosline et al, 2011) These authors observed that *L. donovani* is more sensitive to DTT than macrophages, which reinforces that the ER stress pathway in trypanosomatids would be an interesting target for the development of new chemotherapeutic agents.

The ER-stress response in *T. cruzi* is still poorly understood. Studies from Zingales et al. (1985) and further by Souto-Padrónde Souza (1989) provided the first evidence that treatment of *T. cruzi* with TM might interfere with parasite´s infection of mammalian cells. However, both studies focus on glycosylation of surface proteins rather than in ER-stress response. Thus, motivated by relevant works on the ER stress response in *Leishmania* spp and particularly in *T. brucei*, our group decided to study the effect of TM and DTT on *T. cruzi* epimastigote ultrastructure and expression of UPR-related proteins (Sandes et al., 2019). We have demonstrated that TM treatment resulted in strong growth arrestment at any concentration or time incubation tested. As no signal of cell death by apoptosis or necrosis was observed, it is possible that autophagy is protecting epimastigote from TM-induced ER stress whereas removed and degraded unfunctional organelles or protein aggregates. TM treatment also induced an increase in mRNA levels of both BiP and CRT. Thus, it is reasonable to assume that both chaperones are acting to alleviate the cell stress induced by this drug, although the BiP protein levels remained unchanged (Sandes et al., 2019). TM treatment induced depolarization of the ΔΨm without pronounced ROS production and only few cells (≈10%) presented apoptosis phenotype, which suggests that the autophagy/ER-phagy is the major PCD participating in the TM-induced ER stress response (Sandes et al., 2019). On the other hand, DTT treatment inhibited cell growth, induced drastic morphological changes compatible with cell death. The persistent ER stress induced by DTT seems to target mainly the mitochondrion, inducing drastic morphological changes followed by ΔΨm depolarization and a high generation of mitochondrial ROS. This suggests that the DTT action mechanism is caused mainly by the redox stress rather than the ER stress, as proposed by Tiengwe et al. (2015). The expression of BiP was not affected by DTT treatment, whereas the mRNA levels of BiP and CRT were significantly reduced. Our results suggested that TM induces autophagy/ER-phagy without causing substantial injury to the parasite, whereas the DTT treatment seems to rupture the mitochondrion homeostasis leading to parasite death (Sandes et al., 2019).

## Concluding remarks

Despite the importance of ER for the survival and death of parasitic trypanosomatids, most aspects of its physiology, organization, and response to ER-stress remain unrevealed. Furthermore, compared to *T. brucei*, there is a significant gap in our understanding of ER physiology in other trypanosomatids such as *T. cruzi* and *Leishmania* spp. These limitations have hampered the development of drugs targeting the ER´s pathways in these parasites. In contrast, herein we demonstrated that the ER in trypanosomatids differ from those of mammalian hosts in many aspects, ranging from calcium homeostasis to protein glycosylation, transport, and response to ER stress. For example, mammalian cells rely on three complementary mechanisms to detect and respond to ER stress (IRE1, PERK and ATF6), whereas trypanosomatids, such as *T. brucei*, basically respond to ER stress by the stabilization of m-RNA (short-time stress stimulus) and by Splice Leader Silencing (persisting ER stress). As a result, trypanosomatids may be more susceptible to ER insults induced by drugs than mammalian cells. In line with this hypothesis, unlike host macrophages which have a conventional UPR pathway, the mere existence of the PERK pathway in *L. donovani* makes this parasite particularly vulnerable to DTT-induced stress. Furthermore, TM treatment caused *L. major* apoptosis (Gosline et al., 2011; Dolai et al., 2011 and Peng et al., 2021). In *T. cruzi*, TM triggered autophagy at a low concentration, whereas DTT led to mitochondrial dysfunction, ROS production and cell death (Sandes et al, 2019). When compared to mammalian cells, the cell surface of trypanosomatids contains an unusually high number of GPI-anchored molecules, suggesting that these anchored-proteins provides critical benefits for their adaptation and survival in vertebrate hosts. Thus, compounds that inhibit their biosynthesis, translocation, modification, and transport in the parasite’s ER may have a significant impact on therapeutic development. In this review we showed that SLS is activated by a variety of factors such as depletion of SRP receptor, and perturbations of elements involved in translocation of proteins into de ER, their folding, and modification. PK3 kinase have demonstrated to be essential in this process and its activation during persisting ER stress induces PCD and consequently the parasite elimination. Thus, small molecules that selectively activate PK3 could be considered an attractive drug target (Okalang et al, 2021). Finally, but not least, a better understanding of how the ERQC and UPR work, as well as the mechanisms underlying cell death caused by persistent ER stress in trypanosomatids, can reveal molecular peculiarities, opening new avenues for the development of more effective and less toxic drugs to combat the devastating illness caused by these protozoans.

## Author contributions

RF and JS conceived and wrote the review. All authors contributed to the article and approved the submitted version.

## Funding

This work was supported by Oswaldo Cruz Foundation (FIOCRUZ), Aggeu Magalhães Institute, CNPq (The Brazilian National Council for Scientific and Technological Development -APQ 400749/2019-0). This study was financed in part by the Coordenação de Aperfeiçoamento de Pessoal de Nível Superior-Brasil (CAPES)-Finance Code 001.

## Acknowledgments

The authors would like to thank to Dr. Karina Saraiva and Dr. Cássia Docena for technical assistance and the Program for Technological Development in Tools for Health-PDTISFIOCRUZ for the use of its facilities.

## Conflict of interest

The authors declare that the research was conducted in the absence of any commercial or financial relationships that could be construed as a potential conflict of interest.

## Publisher’s note

All claims expressed in this article are solely those of the authors and do not necessarily represent those of their affiliated organizations, or those of the publisher, the editors and the reviewers. Any product that may be evaluated in this article, or claim that may be made by its manufacturer, is not guaranteed or endorsed by the publisher.
